# Multimorbidity patterns influence mobility disability prevention in frail older adults from the SPRINTT trial

**DOI:** 10.1038/s43587-026-01188-x

**Published:** 2026-08-31

**Authors:** Davide Liborio Vetrano, Caterina Gregorio, Federico Triolo, Luca Soraci, Antonio Cherubini, Matteo Tosato, Stephan von Haehling, Emanuele Marzetti, Francesco Landi, Riccardo Calvani

**Affiliations:** 1 Aging Research Center, Department of Neurobiology, Care Sciences and Society, Karolinska Institutet and Stockholm University, Stockholm, Sweden; 2 Stockholm Gerontology Research Center, Stockholm, Sweden; 3 Unit of Geriatric Medicine and Clinical Epidemiology, Italian National Institute of Health and Sciences on Ageing (INRCA IRCCS), Cosenza, Italy; 4 Geriatria, Accettazione Geriatrica e Centro di Ricerca per l’Invecchiamento, INRCA IRCCS, Ancona, Italy; 5 Department of Clinical and Molecular Sciences (DISCLIMO), Università Politecnica delle Marche, Ancona, Italy; 6 Fondazione Policlinico Universitario ‘A. Gemelli’ IRCCS, Rome, Italy; 7 Department of Geriatrics, Orthopaedics and Rheumatology, Università Cattolica del Sacro Cuore, Rome, Italy; 8 Department of Cardiology and Pneumology, University Medical Centre Göttingen, Georg-August University, Göttingen, Germany; 9 German Centre for Cardiovascular Research (DZHK), Partner Site Göttingen, Göttingen, Germany

**Keywords:** Medical research, Scientific community, Ageing

## Abstract

Preserving independence in older adults is a public health priority. Lifestyle interventions can prevent physical disability, but whether their efficacy is influenced by multimorbidity profiles is unclear. In this post hoc analysis of the SPRINTT randomized trial (NCT02582138), we investigated whether the efficacy of a multicomponent intervention (physical activity with technological support and nutritional counseling) differed across multimorbidity patterns. We included 1,199 community-dwelling adults aged ≥70 years with frailty, sarcopenia and two or more chronic conditions. Latent class analysis identified four multimorbidity patterns: unspecific (55%), psychiatric (24%), cardiometabolic (13%) and respiratory (7%). The intervention reduced the overall risk of mobility disability or death (hazard ratio (HR) 0.78, 95% confidence interval (CI) 0.66–0.92). In subgroup analyses, a significant benefit was observed in participants with a psychiatric multimorbidity pattern (HR 0.65, 95% CI 0.47–0.92). Bayesian partial pooling analyses also supported a beneficial effect in the unspecific pattern (HR 0.80, 95% credible interval 0.69–0.93), while estimates for the cardiometabolic and respiratory patterns were imprecise and not statistically significant. The intervention increased the event-free probability at 24 and 36 months and extended the time free from mobility disability or death by 2.09 months overall. Gains were 3.09 months in the psychiatric pattern and 1.99 months in the unspecific pattern, with no meaningful differences observed in the cardiometabolic and respiratory patterns. These findings suggest that the composition of multimorbidity may influence responsiveness to lifestyle interventions and that stratification based on disease patterns could improve the targeting and effectiveness of preventive strategies in older adults.

## Abstract (web summary)

Lifestyle interventions can preserve the independence of older adults. Here the authors show that multimorbidity patterns influenced the efficacy of a multicomponent lifestyle intervention targeting mobility disability in frail older adults. Participants with psychiatric and unspecific disease patterns experienced the most consistent clinical benefits.

## Main

Globally, the population of older adults is expanding at an unprecedented pace, with those aged 65 years and older representing the fastest-growing demographic group in Europe^1^. While increased longevity is a major public health achievement, a key priority is to ensure that these additional years are spent in good health, preserving functional capacity, independence and quality of life^2^. Preventing functional decline and disability has, therefore, become a central public health objective^2^.

Mobility, defined as the ability to walk independently, is a cornerstone of autonomy in later life^3,4^. Loss of mobility is associated with morbidity, disability and mortality^5^. Operationally, the capacity to walk 400 m is recognized as a valid proxy for community ambulation and a critical prerequisite for maintaining independence^6,7^. Evidence suggests that lifestyle-based interventions can slow or even reverse the progression toward mobility disability in older adults with functional limitations. The Lifestyle Interventions and Independence for Elders (LIFE) randomized trial demonstrated that a structured, moderate-intensity physical activity program significantly reduced the incidence of mobility disability over 2.6 years in physically inactive older adults at risk for disability, compared to health education^8^. Similarly, the Sarcopenia and Physical fRailty IN older people: multicomponenT Treatment strategies (SPRINTT) randomized trial showed that a multicomponent intervention, comprising physical activity with technological support and nutritional counseling, reduced the risk of mobility disability in older adults with physical frailty and sarcopenia, relative to a health education program^9^. Neither trial detected significant heterogeneity in intervention effects across individual disease conditions.

Multimorbidity, typically defined as the co-occurrence of two or more chronic conditions, is highly prevalent in older individuals^10^. Diseases do not codevelop randomly; rather, they tend to cluster within individuals due to shared risk factors, common pathophysiological mechanisms or iatrogenic effects of treatments^10^. Distinct multimorbidity patterns, such as cardiometabolic, neuropsychiatric and musculoskeletal, have been identified, each carrying different implications for functional decline, dementia, disability, institutionalization and survival^11–15^. Hence, these patterns delineate relatively homogeneous subgroups with shared biology and clinical trajectories.

Although the impact of multimorbidity patterns on responsiveness to lifestyle interventions is not well established, preliminary evidence suggests that efficacy may differ across multimorbidity profiles. For example, secondary analyses of exercise trials in community-dwelling and hospitalized frail older adults reported that functional benefits varied by disease pattern^16,17^. Given the heterogeneity inherent in multimorbidity, stratifying individuals according to disease patterns may improve the targeting and effectiveness of preventive interventions. Using data from the SPRINTT trial, we investigated whether multimorbidity patterns modified responses to a multicomponent lifestyle intervention and whether disease clustering influenced intervention responsiveness in frail, sarcopenic older adults with multimorbidity.

## Results

### Participants

**Fig. 1:**
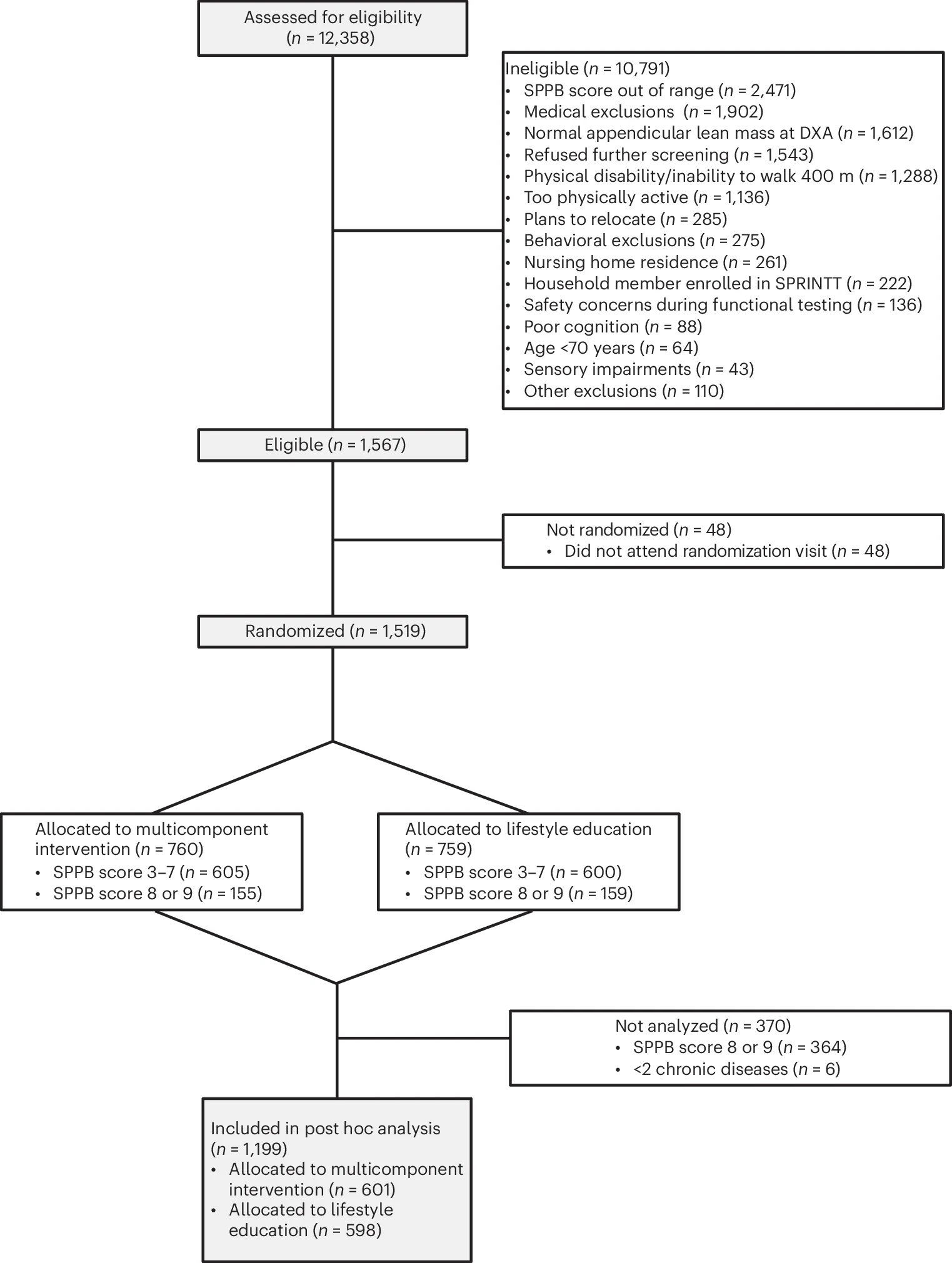
Flowchart of the SPRINTT multimorbidity post hoc analysis. Of the 1,519 randomized participants in the SPRINTT trial, 1,199 with an SPPB score of 3–7 (the primary trial population) and at least two chronic diseases were included in the present post hoc analysis. DXA, dual-energy X-ray absorptiometry.

Of the 1,205 randomized participants with baseline scores on the Short Physical Performance Battery (SPPB)^18^ of 3–7 (the primary trial population), 1,199 with multimorbidity (defined as two or more chronic conditions) were included in this analysis (Fig. 1). The median age was 79.0 years (interquartile range (IQR) 75.0–84.0), and 71% were women. The median number of chronic conditions was 6.0 (IQR 5.0–8.0). Baseline characteristics were comparable between the intervention groups (Table 1).

**Table 1:** Description of study population overall and by intervention group

Characteristics	Overall (*n* = 1,199)^a^	Healthy aging lifestyle education program (*n* = 598)^a^	Multicomponent intervention (*n* = 601)^a^	*P* value^b^
Sex				0.8
Female	855 (71%)	424 (71%)	431 (72%)	
Male	344 (29%)	174 (29%)	170 (28%)	
Age (years)	79.0 (75.0–84.0)	79.0 (75.0–83.0)	79.0 (75.0–84.0)	0.7
Age group				0.6
70–74	283 (24%)	138 (23%)	145 (24%)	
75–79	367 (31%)	196 (33%)	171 (28%)	
80–84	293 (24%)	138 (23%)	155 (26%)	
85–89	196 (16%)	96 (16%)	100 (17%)	
≥90	60 (5.0%)	30 (5.0%)	30 (5.0%)	
Number of chronic conditions	6.00 (5.00–8.00)	6.00 (5.00–8.00)	6.00 (5.00–8.00)	0.4
Multimorbidity pattern				0.9
Unspecific	660 (55%)	330 (55%)	330 (55%)	
Respiratory	88 (7.3%)	40 (6.7%)	48 (8.0%)	
Psychiatric	290 (24%)	147 (25%)	143 (24%)	
Cardiometabolic	161 (13%)	81 (14%)	80 (13%)	

^a^Data are presented as *n* (%) or median (IQR).

^b^*P* values were calculated using Pearson’s chi-squared test or the Wilcoxon rank-sum test.

### Multimorbidity patterns

**Fig. 2:**
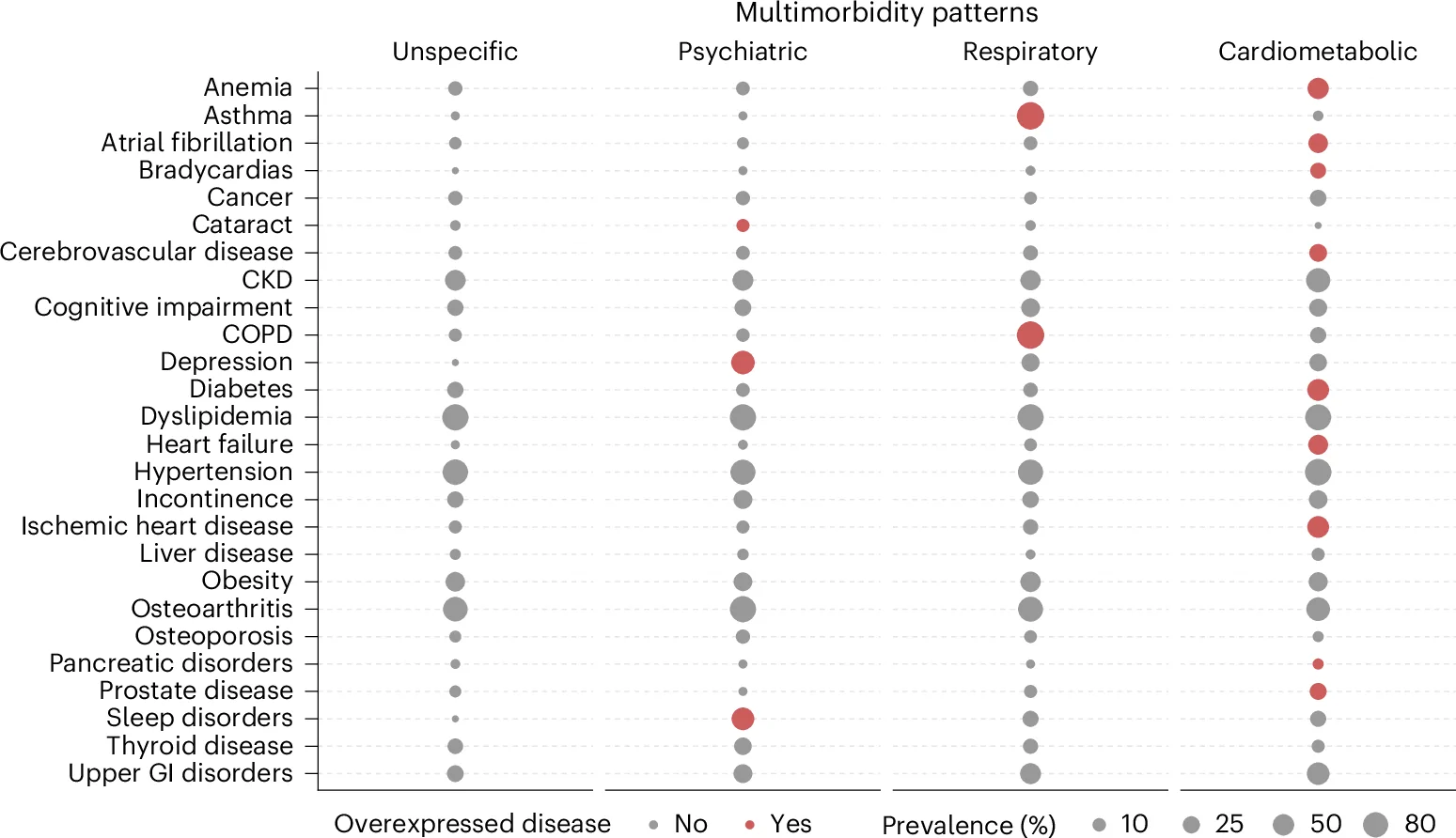
Bubble plot depicting the prevalence of chronic diseases in each of the multimorbidity patterns. Red bubbles indicate diseases considered overexpressed within each multimorbidity pattern (unspecific, *n* = 660; respiratory, *n* = 88; psychiatric, *n* = 290; cardiometabolic, *n* = 161). Overexpression is defined by an observed-to-expected ratio of 2 or greater and an exclusivity of at least 25%, indicating that the disease is both disproportionately prevalent and relatively unique to that pattern. CKD, chronic kidney disease; COPD, chronic obstructive pulmonary disease; GI, gastrointestinal.

We identified four multimorbidity patterns through latent class analysis (LCA) (Supplementary Fig. 1): unspecific (no overexpressed diseases; 660 participants, 55%), respiratory (88 participants, 7%), psychiatric (290 participants, 24%) and cardiometabolic (161 participants, 13%). Overexpressed diseases across identified multimorbidity patterns are shown in a bubble plot in Fig. 2, while their prevalence and distribution are reported in Supplementary Table 2. Multimorbidity patterns were evenly distributed between the intervention groups (*P* = 0.9).

**Fig. 3:**
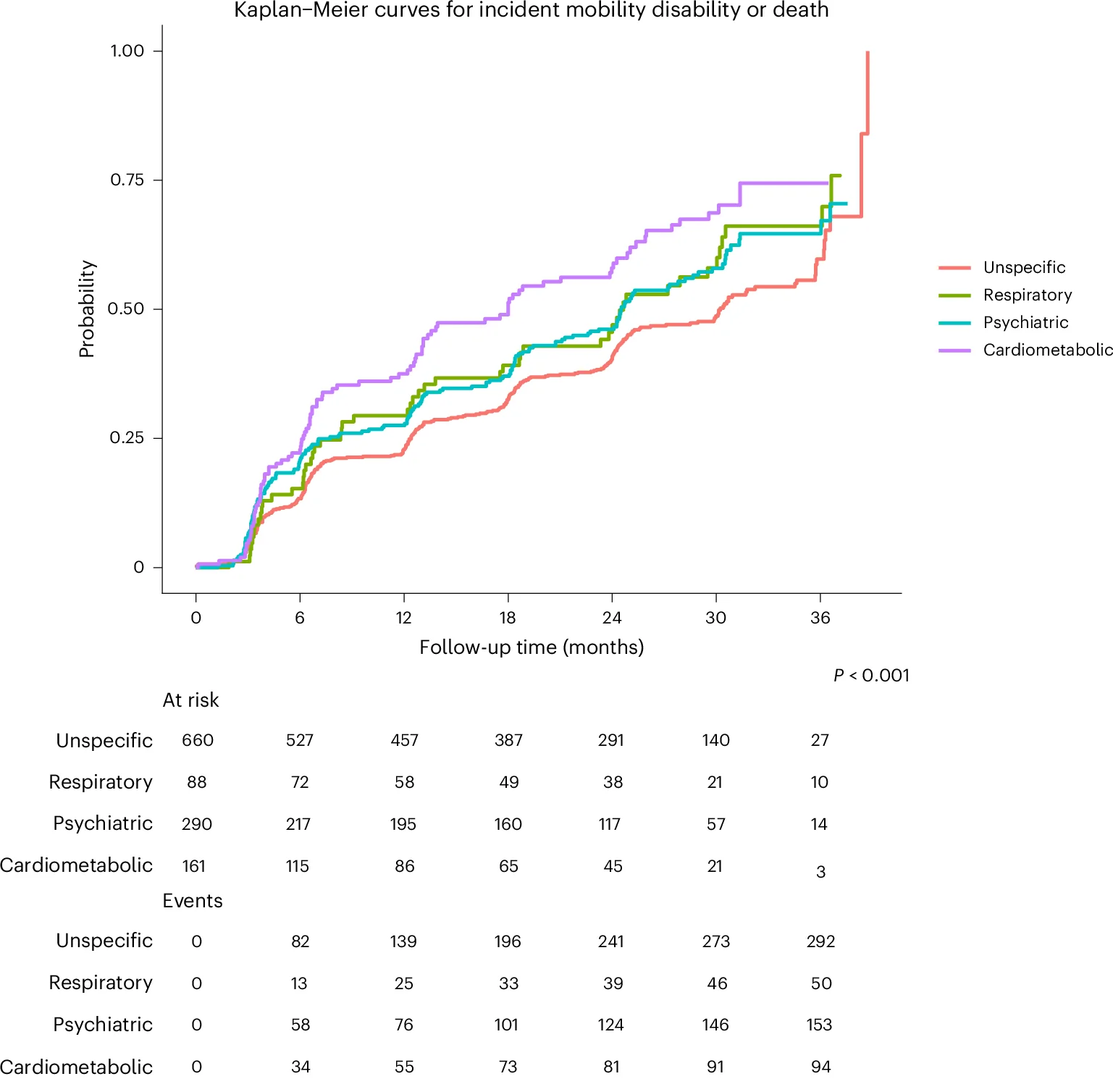
Kaplan–Meier incidence curves for the primary endpoint stratified by multimorbidity patterns. The incidence curves differed significantly across multimorbidity patterns (*P* < 0.001, two-sided log-rank test), rejecting the null hypothesis that the probability of incident mobility disability or death was identical across all groups.

Significant differences were observed across multimorbidity patterns in sex, age and disease burden (Table 2). Those in the psychiatric group were predominantly women (89%), whereas the cardiometabolic group included more men (57%) and had the highest median age (82 years; IQR 77–86). Participants in the unspecific group had the lowest disease burden (median 5 conditions; IQR 4–6), while the cardiometabolic group had the highest disease burden (median 9 conditions; IQR 7–10). Cumulative incidence of mobility disability or death differed significantly across patterns (Fig. 3; *P* = 0.007), with higher event rates in the cardiometabolic (*P* = 0.04; 178 events per person-year) and psychiatric (*P* = 0.001; 130 events per person-year) groups compared to the unspecific group (105 events per person-year). The respiratory pattern (137 events per person-year) showed a similar event rate to the psychiatric pattern.

**Table 2:** Description of study population overall and by multimorbidity pattern

Characteristics	Overall (*n* = 1,199)^a^	Unspecific (*n* = 660)^a^	Respiratory (*n* = 88)^a^	Psychiatric (*n* = 290)^a^	Cardiometabolic (*n* = 161)^a^	*P* value^b^
Sex						<0.001
Female	855 (71%)	460 (70%)	67 (76%)	259 (89%)	69 (43%)	
Male	344 (29%)	200 (30%)	21 (24%)	31 (11%)	92 (57%)	
Age (years)	79.0 (75.0–84.0)	79.0 (74.8–83.0)	78.0 (74.0–81.0)	78.0 (74.0–83.0)	82.0 (77.0–86.0)	<0.001
Age group						<0.001
70–74	283 (24%)	165 (25%)	23 (26%)	74 (26%)	21 (13%)	
75–79	367 (31%)	189 (29%)	37 (42%)	99 (34%)	42 (26%)	
80–84	293 (24%)	178 (27%)	12 (14%)	64 (22%)	39 (24%)	
85–89	196 (16%)	100 (15%)	15 (17%)	39 (13%)	42 (26%)	
≥90	60 (5.0%)	28 (4.2%)	1 (1.1%)	14 (4.8%)	17 (11%)	
Number of chronic diseases	6.00 (5.00–8.00)	5.00 (4.00–6.00)	8.00 (7.00–10.00)	7.00 (5.25–8.00)	9.00 (7.00–10.00)	<0.001
Number of drugs	5 (3–7)	4 (3–6)	6 (4–9)	5 (3–7)	8 (6–10)	<0.001
SPPB score	7.00 (6.00–7.00)	7.00 (6.00–7.00)	6.00 (5.00–7.00)	7.00 (6.00–7.00)	6.00 (5.00–7.00)	0.021
MMSE score	28.00 (27.00–29.00)	28.00 (27.00–29.00)	28.00 (26.00–29.00)	28.00 (27.00–29.00)	28.00 (26.00–29.00)	0.023
Current smoking	68 (5.8%)	40 (6%)	7 (8.0%)	18 (6.2%)	3 (1.9%)	0.20
Missing	19	14	0	0	5	
Drinking alcohol in the last 12 months	441 (40%)	238 (40%)	30 (37%)	102 (38%)	71 (48%)	0.2
Missing	97	61	6	18	12	
Living alone	595 (50%)	325 (50%)	42 (48%)	162 (56%)	66 (42%)	0.044
Missing	17	13	0	0	4	
Low education (none or elementary)	285 (25%)	165 (26%)	23 (27%)	65 (23%)	32 (21%)	0.6
Missing	47	23	2	11	11	
Manual occupation	323 (30%)	173 (28%)	37 (45%)	76 (30%)	37 (25%)	0.010
Missing	112	52	6	40	14	

^a^Data are presented as *n* (%), median (IQR) and counts of missing values.

^b^*P* values were calculated using Pearson’s chi-squared test or the Kruskal–Wallis rank-sum test.

### Inverse probability of treatment weighting

Inverse probability of treatment weighting (IPTW) was applied to ensure balance between treatment arms within each multimorbidity pattern using all baseline covariates listed in Table 2. As shown in Supplementary Fig. 2, all patterns exhibited some degree of initial imbalance; however, IPTW substantially improved balance across covariates. After weighting, covariate balance improved substantially across multimorbidity patterns, with most absolute standardized differences reduced to <0.1 and nearly all <0.2, indicating acceptable balance for the weighted analyses.

### Efficacy endpoints

**Fig. 4:**
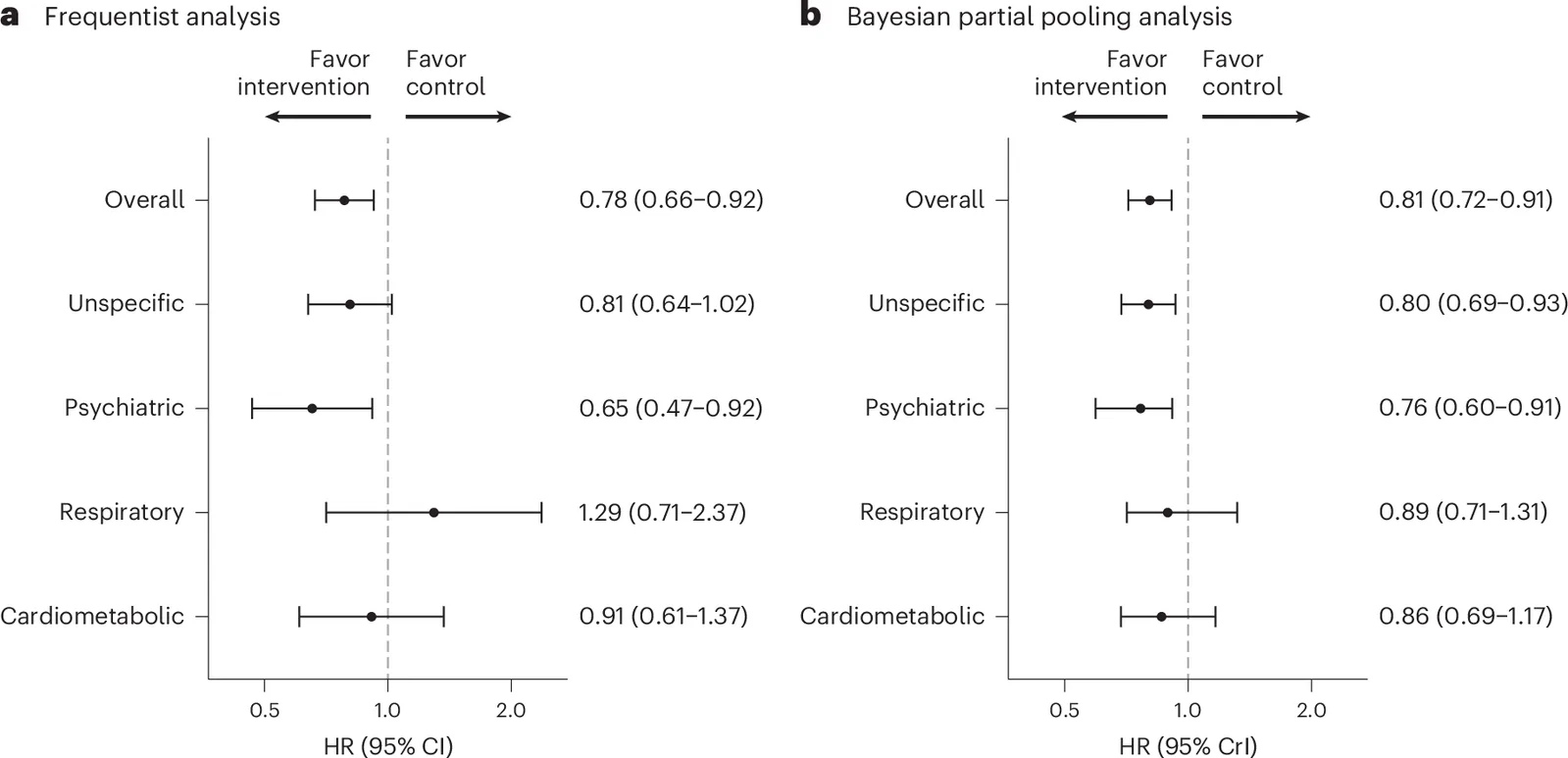
Hazard ratios (HRs) of the intervention effect with 95% confidence intervals (CIs, frequentist) and 95% credible intervals (CrIs, Bayesian) for the overall population (*n* = 1199) and each multimorbidity pattern (unspecific, *n* = 660; respiratory, *n* = 88; psychiatric, *n* = 290; cardiometabolic, *n* = 161). In both panels, the central point denotes the estimated HR, and the horizontal error bars represent the corresponding 95% CI (**a**) or 95% CrI (**b**). In **a**, HRs were estimated from separate IPTW-adjusted Cox proportional hazards models stratified by sex and study site. In **b**, the corresponding estimates were derived from an IPTW-adjusted Bayesian partial pooling Cox model, which shrinks subgroup-specific estimates toward the overall effect based on the amount of information available within each multimorbidity pattern. HRs <1 indicate a protective effect of the intervention.

After adjustment for multimorbidity patterns, participants in the multicomponent intervention group had a significantly lower risk of mobility disability than those in the health education group (hazard ratio (HR) 0.78, 95% confidence interval (CI) 0.66–0.92). Statistical interaction between the intervention and the multimorbidity patterns was not significant (*P* = 0.870). Nevertheless, based on the high biological plausibility of effect modification by multimorbidity patterns on the intervention, we performed subgroup analyses according to these patterns. These analyses were carried out using two different approaches: a classic frequentist approach and a recently proposed Bayesian method to estimate subgroup effects from intervention studies^19^. In accordance with the frequentist approach, the only statistically significant reduction in mobility disability was observed among participants displaying a psychiatric pattern (HR 0.65, 95% CI 0.47–0.92; Fig. 4a and Supplementary Fig. 3). The Bayesian partial pooling analysis yielded broadly consistent findings, with posterior HRs indicating a protective effect of the intervention in the unspecific (HR 0.80, 95% credible interval (CrI) 0.69–0.93) and psychiatric (HR 0.76, 95% CrI 0.60–0.91) multimorbidity patterns, while the respiratory and cardiometabolic patterns showed more heterogeneous and less precise estimates (Fig. 4b). The posterior probability that the intervention is beneficial (HR < 1) was 0.998 for the unspecific pattern, 0.998 for the psychiatric pattern, 0.851 for the cardiometabolic pattern and 0.736 for the respiratory pattern, further supporting strong evidence of benefit in the unspecific and psychiatric groups and more uncertain evidence in the remaining patterns.

At 24 months, the event-free probability was 7.5% higher in the multicomponent intervention group than in the health education group (absolute difference 0.075, 95% CI 0.03–0.12), increasing to 8.2% at 36 months (absolute difference 0.082, 95% CI 0.033–0.131). At 12 months, the corresponding absolute difference was 5.3% (0.053, 95% CI 0.01–0.10). Over 36 months, participants assigned to the multicomponent intervention remained free from mobility disability or death for an average of 2.09 months longer (95% CI 0.86–3.33 months) than those in the health education group.

**Fig. 5:**
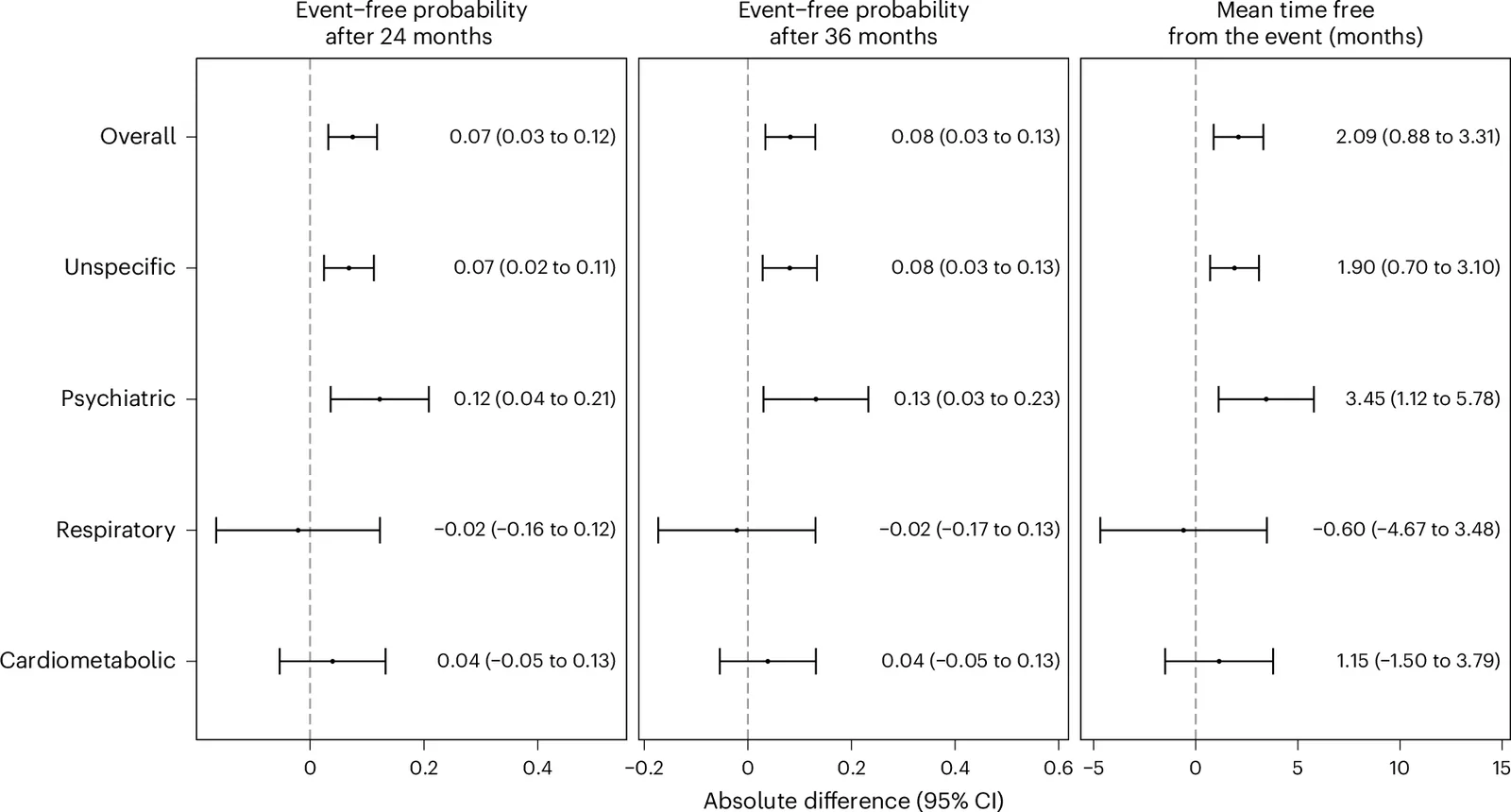
Absolute measures of effect in overall (*n* = 1199) and in subgroup analyses by multimorbidity patterns (unspecific *n* = 660; respiratory *n* = 88; psychiatric *n* = 290; cardiometabolic *n* = 161) with 95% confidence intervals (CIs). In all panels, the central point denotes the estimated absolute treatment effect, and the horizontal error bars represent the corresponding 95% CI. The absolute differences between the two intervention groups in the event-free probability of the primary endpoint at 24 and 36 months, and in the 36-month restricted mean time free from mobility disability or death, together with the corresponding 95% CIs, were estimated using separate Royston–Parmar flexible parametric survival models in the overall population and within each pattern.

When analyzed by multimorbidity pattern, consistent benefits of the intervention were observed among participants with the unspecific pattern, with absolute increases in event-free probability of 4.7% at 12 months (0.047, 95% CI 0.007–0.086), 6.8% at 24 months (0.068, 95% CI 0.025–0.112) and 8.1% at 36 months (0.081, 95% CI 0.029–0.133). These gains translated into 1.90 additional event-free months over 36 months (95% CI 0.73–3.07 months). A larger effect was seen in the psychiatric pattern, where absolute differences reached 8.8% at 12 months (0.088, 95% CI 0.016–0.159), 12.2% at 24 months (0.122, 95% CI 0.036–0.208) and 13.1% at 36 months (0.131, 95% CI 0.031–0.231). Participants in this group remained event-free for 3.45 months longer over 36 months (95% CI 1.14–5.77 months). In contrast, no meaningful benefit was observed for the respiratory and cardiometabolic patterns, for which the absolute differences at 12, 24 and 36 months were close to zero and not statistically significant (Fig. 5).

In the simulation analysis, the power to detect a treatment-by-pattern interaction increased steadily with sample size, surpassing 80% between 6,000 and 7,000 participants (Supplementary Fig. 4). Because the simulation assumptions were derived from the observed data structure and estimated interaction effects, these findings should be interpreted as contextual rather than confirmatory. Nevertheless, the results suggest that the SPRINTT sample (*n* = 1,199) may have had limited power to detect interaction effects of the magnitude observed in the present study through formal interaction testing or Bonferroni-corrected subgroup-specific analyses. The false-positive rate remained close to the nominal 5% level, as expected.

## Discussion

In this post hoc analysis of the SPRINTT trial, we found that the efficacy of a multicomponent lifestyle intervention in preventing mobility disability among community-dwelling older adults with physical frailty and sarcopenia varied according to multimorbidity patterns. Participants with unspecific and psychiatric patterns of multimorbidity experienced 20% and 24% reductions, respectively, in the risk of the composite outcome, which included mobility disability and death. These participants also demonstrated higher event-free probabilities at 24 and 36 months, as well as 2 and 3.5 additional months free from mobility disability, compared to those with other multimorbidity patterns.

The LIFE^8^ and SPRINTT^9^ trials showed that an exercise-based intervention and a multicomponent lifestyle intervention, respectively, reduced incident mobility disability in vulnerable older adults, with no clear evidence of effect modification across conventional subgroups (for example, age, sex, ethnicity/race, presence of cardiovascular disease or diabetes, and baseline 4-m gait speed). Our analysis extends these findings by demonstrating that disease clustering, rather than single conditions or broad demographic factors, may significantly modify responsiveness to intervention.

This interpretation aligns with prior studies. Martínez-Velilla et al.^17^ reported heterogeneous responses to an exercise intervention across multimorbidity profiles in hospitalized frail older adults. The metabolic multimorbidity pattern, similar in composition to our unspecific multimorbidity pattern, showed the greatest gains in physical function and cognition, whereas the psychiatric pattern was associated with marked improvements in quality of life and depressive symptoms but only modest functional benefits^17^. Further supporting the concept of multimorbidity-driven effect modification, Corica et al.^20^, in a secondary analysis of the Mobile Health (mHealth) Technology to Improve Care for Patients with Atrial Fibrillation (mAFA-II) cluster randomized trial, evaluated an integrated care pathway in patients with atrial fibrillation grouped as low morbidity, hypertension/coronary artery disease or mixed high morbidity. They observed substantial heterogeneity in intervention benefits, with patients in the milder multimorbidity groups achieving the greatest relative risk reduction in the composite outcome of all-cause death, stroke/thromboembolism and rehospitalization^20^. Together, these findings suggest that milder multimorbidity clusters—typically involving fewer organ systems, lower systemic inflammation and greater physiological reserve—are more amenable to preventive interventions. Our results, showing the efficacy of the intervention in participants displaying an unspecific pattern of multimorbidity (often referred to as mild multimorbidity), confirm this hypothesis. In addition, psychiatric conditions, although disabling, often coexist with relatively preserved physical systems, allowing greater potential for improvement through lifestyle-based strategies. This potential is further enhanced when the intervention also improves mental health (for example, motivation, mood), which in turn promotes better adherence to the program^21–23^. Consistent with this, a secondary analysis of the LIFE trial showed that, while depressive symptoms independently increased the risk of mobility disability, they did not modify intervention effects, indicating that older adults with depression benefit comparably from exercise in reducing disability risk^24^. By contrast, multimorbidity profiles such as the cardiometabolic and respiratory patterns are characterized by greater systemic inflammation, vascular dysfunction and multisystem impairment^25–28^, which may limit intervention responsiveness and increase susceptibility to iatrogenic effects or decompensation during follow-up^16,29,30^.

Collectively, these studies and our own analysis indicate that, while multidomain interventions confer overall benefits in older adults with multimorbidity, their effectiveness is contingent upon disease architecture. This supports a precision approach to multimorbidity care, whereby stratifying individuals by patterns—both in experimental and clinical contexts—may enhance targeting, improve efficiency and ultimately reduce the burden of disability in aging populations.

This study has several limitations. First, as a post hoc analysis of a randomized clinical trial, it may have been underpowered to detect certain associations or interactions. As shown in our simulation analysis, larger samples could have strengthened statistical power and revealed subtler differences across multimorbidity patterns. Second, the identification of patterns was based on diagnostic information available within SPRINTT, which had limited coding granularity. Third, generalizability is constrained by the inclusion criteria of SPRINTT, which targeted older adults aged ≥70 years with physical frailty and sarcopenia, underscoring the need for replication in broader populations. Finally, although we hypothesized that multimorbidity patterns may reflect distinct biological substrates, this could not be tested due to a lack of biomarker data. Future studies integrating biological, functional and clinical measures are needed to validate and expand upon these findings.

In summary, the efficacy of a multicomponent intervention in preventing mobility disability differed across multimorbidity patterns. Participants with milder multimorbidity profiles, particularly those in the psychiatric and unspecific groups, derived the greatest benefit. These results suggest the value of multimorbidity-stratified strategies in tailoring preventive interventions for older adults. Future trials should incorporate multimorbidity profiling to refine targeting and maximize clinical impact.

## Methods

### Ethics

The SPRINTT study protocol was approved by the Ethics Committee of Università Cattolica del Sacro Cuore, Rome, Italy (protocol no. 15611/15), which served as the coordinating ethics committee for the trial. The protocol was subsequently ratified by the institutional ethics committees of all participating centers across the 11 European countries involved in the study. The trial was conducted in accordance with the ethical principles of the Declaration of Helsinki and the applicable national and international regulations governing clinical research. All participants provided written informed consent before enrollment and prior to the initiation of any study-related procedures.

### Study design

This post hoc analysis is based on the SPRINTT randomized clinical trial, a multicenter study conducted across 11 European countries that evaluated whether a multicomponent program—including individualized physical activity, nutritional counseling and technology-assisted activity monitoring—could prevent mobility disability in older adults with frailty and sarcopenia^9,31^. Full details of the study protocol are available at ClinicalTrials.gov (NCT02582138) and in a dedicated publication^32^.

### Study population

Participants were community-dwelling adults aged ≥70 years with a newly conceptualized predisability condition termed physical frailty and sarcopenia^31^. The condition of interest was operationalized based on an SPPB score of 3–9 points combined with low appendicular lean mass according to the Foundation for the National Institutes of Health cutpoints^33^, in the absence of mobility disability. Mobility disability was defined as the inability to complete a 400-m walk within 15 min without sitting, pausing for >1 min, requiring assistance or using walking aids other than a single straight cane (for example, tripods, walkers)^34^. Individuals were excluded if they had conditions preventing active and safe participation in trial activities, such as self-reported mobility disability, cognitive impairment (a Mini-Mental State Examination (MMSE)^35^ score of <24), terminal illness, current participation in structured exercise programs, medical contraindications as judged by study physicians or planned relocation outside the study area within the following 2 years. Eligible participants were randomly assigned (1:1) to either the multicomponent intervention group or the healthy aging lifestyle education group using a secure web-based randomization system with permuted blocks, stratified by study site, sex and baseline SPPB score (3–7 versus 8 or 9). Outcome assessors were blinded to treatment allocation, clinical and laboratory measurements, and intervention adherence.

For this analysis, we included randomized participants with baseline SPPB scores of 3–7 (the primary trial population) and with multimorbidity (defined as two or more chronic diseases). Because the analytical sample was inherited from the parent trial, no separate sample size calculation was performed. Recruitment occurred between January 2016 and November 2017; randomization began on February 3, 2016; and the last follow-up visit was on October 31, 2019. Details of screening, recruitment and baseline characteristics have been reported previously^9,36^.

### Intervention

Participants assigned to the multicomponent intervention received a program comprising structured physical activity, personalized nutritional counseling and technology-assisted support. The physical activity component included aerobic, resistance, flexibility and balance training, individually tailored and delivered through supervised center-based sessions (up to four times per week) and home exercises. Nutritional counseling focused on optimizing protein and energy intake, with supplementation provided when necessary^37^. A thigh-worn actimeter was used to monitor adherence to physical activity, provide feedback and enhance motivation. The healthy aging lifestyle education (control intervention) group attended monthly or twice-monthly educational sessions on healthy aging, each followed by simple stretching exercises. Both interventions were delivered for up to 36 months, depending on the enrollment date. Full details of the intervention procedures are reported elsewhere^32^.

### Primary outcome

The primary outcome was incident mobility disability, assessed using the 400-m walk test^34,38^. Participants were instructed to complete ten laps of a 20-m course at their usual pace, avoiding overexertion. The test was administered 3 months after randomization and then every 6 months from the baseline assessment. As specified in the main study^9^, when the test could not be performed, a predefined adjudication process was applied: mobility disability was assigned if gait speed over 4 m was ≤0.4 m s^−1^, if gait speed was >0.4 m s^−1^ with the use of a walking aid other than a single cane, or if walking disability was documented in medical records or reported by the participant or a proxy. Cases not meeting these criteria were reviewed by an independent adjudication committee using clinical data, functional assessments and adverse event reports. Adjudication was not an alternative definition of disability but a method to determine status when the 400-m test was unavailable, integrating observed and adjudicated outcomes to minimize missing endpoint data. Participants were censored at their last successful test if the criteria for disability were not met at trial end, at the first visit with a >9-month gap between two successful tests or at randomization if no postbaseline test was available. The 9-month threshold (prespecified) reflects the 6-month test schedule, with longer gaps indicating missed visits and uncertain timing. Therefore, participants were censored at the last reliable visit, with events occurring after gaps conservatively backdated. Potential informative censoring was partially addressed through adjudication and conservative handling procedures. Mobility disability was defined as occurring on the date a participant failed the 400-m walk test, was unable or unwilling to attempt it and was adjudicated as having the disability, was unable to attempt it without adjudication, or died.

### Disease assessment

Chronic disease diagnoses were established through a standardized assessment integrating multiple data sources. Information was collected through physician-administered medical history interviews (including patient- or proxy-reported diagnoses); structured questionnaires (for example, MMSE, Center for Epidemiologic Studies Depression Scale^39^); anthropometric measurements such as body mass index; vital signs such as blood pressure; and laboratory analyses (for example, fasting glucose, cholesterol, creatinine, hemoglobin). Medication inventories, coded using the Anatomical Therapeutic Chemical classification, were reviewed to support diagnostic adjudication when prescriptions clearly indicated a specific condition. Diagnoses were coded using the International Classification of Diseases, Ninth Revision, and aggregated into 60 chronic disease categories according to a validated, clinically driven classification system^40^. Only baseline conditions were included in the analysis. Further details on disease assessment and coding are provided in Supplementary Table 1.

### Covariates

Demographic variables, including age and sex, were collected during the prerandomization screening. Age was categorized into 5-year groups (that is, 70–74, 75–79, 80–84, 85–89 and ≥90 years). The number of chronic conditions for each participant was derived from the diagnoses classified using the methodology described above.

### Statistics and reproducibility

Baseline characteristics of participants in the two intervention arms are reported as median (IQR) for continuous variables and as number (percentage) for categorical variables. Chronic conditions with a prevalence of ≥2% were used to identify homogeneous multimorbidity groups through LCA^41^. In this context, the term homogeneous indicates that individuals grouped within the same class tend to share similar combinations of chronic conditions. The optimal number of patterns was determined using the adjusted Bayesian information criterion and theoretical interpretability. Each pattern was labeled based on the distinctive set of chronic diseases that were disproportionately represented (that is, overexpressed) within that pattern. Chronic conditions were considered for pattern characterization and labeling if they were overexpressed in a given pattern. Overexpression was defined as an observed-to-expected ratio of ≥2 and an exclusivity of ≥25%. The observed-to-expected ratio was calculated by dividing the prevalence of a given disease within a specific pattern by its prevalence in the overall multimorbid population, while exclusivity was calculated by dividing the absolute frequency of that disease within the specific pattern by its frequency in the overall multimorbid population. Although each pattern is defined by a distinct combination of chronic conditions, it may also include additional conditions that are present but not prominently expressed. Each participant was assigned to the pattern with the highest posterior probability of membership^42^.

The primary endpoint (time to first occurrence of mobility disability or death) was compared across multimorbidity patterns and between intervention groups using two-sided log-rank tests at the 5% *α*-level, stratified by randomization factors of study site and sex.

To account for baseline differences between the intervention and control groups within each multimorbidity pattern, all subgroup analyses were conducted using IPTW. Propensity scores were estimated using generalized boosted models to flexibly model the treatment assignment mechanism and iteratively optimize covariate balance. In estimating the weights, we targeted the average treatment effect, ensuring that the weighted sample represents the entire study population rather than only the treated or untreated individuals. The boosting algorithm was guided by the ‘es.mean’ stopping rule, which selects the iteration that minimizes the mean absolute standardized difference across covariates. This configuration directs the algorithm to select the propensity score model that minimizes the mean standardized effect size across covariates, thereby improving balance without requiring a priori specification of nonlinearities or interactions. Covariate balance was evaluated within each multimorbidity pattern using the absolute standardized difference for all baseline variables before and after weighting.

Cox proportional hazards models, also stratified by site and sex, were used to estimate HRs with 95% CIs, additionally adjusting for multimorbidity patterns. Sandwich robust standard errors were used to account for the uncertainty introduced by the weighting procedure and to ensure valid inference under the IPTW framework. The proportional hazards assumption was assessed using Schoenfeld residuals by testing for independence between the residuals and time. Cumulative incidence functions were estimated using the Kaplan–Meier method.

To further characterize intervention effects, Royston–Parmar flexible parametric survival models were applied to estimate the event-free probability of the primary endpoint at 24 and 36 months, as well as the 36-month restricted mean time free from mobility disability or death, with corresponding 95% CIs. In these models, the log cumulative hazard was modeled as a natural cubic spline, with degrees of freedom selected according to the Bayesian information criterion. Subgroup analyses using Cox and flexible parametric models assessed whether intervention effects varied according to multimorbidity patterns.

To complement the frequentist analyses, we implemented a Bayesian partial pooling framework based on the Cox proportional hazards model^19^. For each simulation scenario, study-specific log HRs were modeled as hierarchical random effects drawn from a common population distribution. We assumed weakly informative priors for both the fixed effects and the between-study variance, allowing the amount of shrinkage to adapt to the information available in each study. Posterior inference was obtained using Markov chain Monte Carlo sampling, and pooled treatment effects were estimated as the posterior means of the study-level parameters. In this Bayesian framework, 95% CrIs are obtained as quantiles of the posterior distribution of the parameters and represent the probability that the true subgroup-specific HRs lie within the interval, given the observed data and model. This approach formally accounts for heterogeneity while borrowing strength across studies, reducing instability in scenarios involving uneven subgroup distributions or small sample sizes. It was not possible to implement a Bayesian hierarchical (partial pooling) analog of the Royston–Parmar flexible parametric model comparable to the frequentist implementation. Therefore, results for the Royston–Parmar model are limited to frequentist analyses only.

We conducted an exploratory Monte Carlo simulation to provide contextual information on the statistical power to detect treatment-by-pattern interaction effects under the empirical conditions of the SPRINTT trial. The data-generating mechanism was based on the fitted Cox proportional hazards model: the empirical baseline cumulative hazard was extracted and inverted to simulate event times, and covariates were generated to match the observed multimorbidity pattern distribution and treatment allocation. In the primary simulated scenario, the data-generating mechanism incorporated the treatment-by-pattern interaction coefficients estimated in the trial; in a complementary scenario, these coefficients were set to zero to quantify the false-positive rate. For each scenario and for sample sizes ranging from *n* = 1,199 to 10,000, 1,000 datasets were generated, and Cox models were fitted to estimate power, false-negative probability and false-positive probability.

All analyses were conducted in R (version 4.5.2) using the following packages: poLCA to derive latent multimorbidity patterns, twang for generalized boosted models and propensity score weighting, survival for Cox proportional hazards models, flexsurv for Royston–Parmar flexible parametric models, brms for Bayesian hierarchical modeling and ggplot for graphics.

### Reporting summary

Further information on research design is available in the Nature Portfolio Reporting Summary linked to this article.

## Supplementary information

Supplementary Information Supplementary Tables 1 and 2 and Figs. 1–4.

Reporting Summary

## Data Availability

The SPRINTT trial data are not publicly available due to participant confidentiality and data protection requirements. Deidentified individual participant data underlying the results reported in this article may be requested from L. Mariotti (luca.mariotti1@unicatt.it). Requests will be reviewed to ensure compliance with participant confidentiality, ethical approvals and intellectual property requirements. A data-sharing agreement must be signed before the data are released.

## Code availability

No custom code or mathematical algorithm was used in the study. Analysis scripts for this study are available from the corresponding author upon request.
