# Supplementary material for: Multimorbidity patterns influence mobility disability prevention in frail older adults from the SPRINTT trial
Source: Nat Aging. 2026 Aug 31;6(9):1967–78. doi: 10.1038/s43587-026-01188-x (PMC13577888; doi:10.1038/s43587-026-01188-x)
Supplement: Supplementary file 1 [file 43587_2026_1188_MOESM1_ESM.pdf]

nature aging

Supplementary information

https://doi.org/10.1038/s43587-026-01188-x

Multimorbidity patterns influence mobility disability prevention in frail older adults from the SPRINTT trial

In the format provided by the authors and unedited

Table of contents

Supplementary table 1. Chronic disease assessment and coding. 2

Supplementary table 2. Chronic disease prevalence and distribution of the number of chronic diseases in the study population across multimorbidity patterns.. 5

Supplementary fig. 1. Adjusted Bayesian information criteria (ABIC) across different number of latent classes 7

Supplementary fig. 2. Covariate balance before and after inverse probability of treatment weighting (IPTW) across multimorbidity patterns (unspecific, n=660; respiratory, n=88; psychiatric, n=290; cardiometabolic, n=161) 8

Supplementary fig. 3. Inverse probability of treatment weighting (IPTW) Kaplan−Meier curves for incident mobility disability or death within each of the multimorbidity patterns 9

Supplementary fig. 4. Power to detect treatment–pattern interaction across increasing sample sizes in the Monte Carlo simulation calibrated to multimorbidity pattern subgroup analyses in the SPRINTT trial 10

Supplementary table 1. Chronic disease assessment and coding.

Condition	Definition/Source
Allergy	Self or proxy-reported diagnosis, including "allergic rhinitis", "eye allergy", "eczema"
Anaemia	Self or proxy-reported diagnosis, including "iron deficiency anaemia", "vitamin B12 deficiency anaemia", "macrocytic anaemia", "haemorrhagic anaemia", "megaloblastic anaemia"<br>Haemoglobin values <13mg/dL for men and <12mg/dL for women<br>Use of antianaemic preparations (ATC code: B03)
Arteriosclerosis	Self or proxy-reported diagnosis, including "carotid arteriosclerosis", "aneurysm", or procedure such as "carotid endarterectomy"
Asthma	Self or proxy-reported diagnosis
Atrial fibrillation	Self or proxy-reported diagnosis<br>Use of vitamin K antagonists (B01AA), direct thrombin and factor Xa inhibitors (B01AE and B01AF)
Autoimmune diseases	Self or proxy-reported diagnosis
Blindness/visual impairment	Self or proxy-reported diagnosis
Bradycardia	Self or proxy-reported diagnosis, including "bradycardia", "atrioventricular block", "cardiac conduction disorders", or pacemaker implantation procedure
Cancer	Self or proxy-reported diagnosis, including "breast, prostate and colon cancer", "lymphoma", or mastectomy procedure
Cardiac valve disease	Self or proxy-reported diagnosis, including "cardiac valve disease" or "cardiac valve disorders", or procedures including "aortic valve replacement", "valvuloplasty", "mitral valve replacement", "mitral valve operation", "transcatheter aortic valve implantation"
Cataract	Self or proxy-reported diagnosis
Cerebrovascular disease	Self or proxy-reported diagnosis of "stroke" or "transient ischemic attack"
Chronic kidney disease	Self or proxy-reported diagnosis<br>Estimated glomerular filtration rate (eGFR) <60 ml/min using Berlin initiative study equation[2,3]
Chronic obstructive pulmonary disease	Self or proxy-reported diagnosis<br>Use of drugs for obstructive airway disease (R03)
Cognitive impairment	Total score at mini mental state examination[4] ≤26
Deafness/hypoacusis	Self or proxy-reported diagnosis<br>Use of hearing aids
Depression	Total score at modified Center for Epidemiologic Studies Depression Scale (11 items) [5] ≥9
Diabetes	Self or proxy-reported diagnosis

	Use of drugs used in diabetes (A10)
Dyslipidaemia	Self or proxy-reported diagnosis including “dyslipidaemia”, “hyperlipidaemia”, hypercholesterolaemia”<br><br>Blood total cholesterol ≥200 mg/dL, or LDL cholesterol >100 mg/dL, or HDL cholesterol <40 mg/dL (men) or triglycerides >150 mg/dL<br><br>Use of HMG CoA reductase inhibitors (C10AA) or other lipid modifying agents (C10AX)
Epilepsy	Self or proxy-reported diagnosis
Glaucoma	Self or proxy-reported diagnosis
Gout	Self or proxy-reported diagnosis
Heart failure	Self or proxy-reported diagnosis
Hypertension	Self or proxy-reported diagnosis, including “hypertension” or “high blood pressure”<br><br>Systolic blood pressure ≥140 mmHg or diastolic blood pressure ≥90 mmHg<br><br>Use of antihypertensives (C02)
Inflammatory bowel disease	Self or proxy-reported diagnosis, including “ulcerative colitis”, “inflammatory bowel disease”, “Crohn’s disease”
Ischemic heart disease	Self or proxy-reported diagnosis, including “heart attack, coronary, or myocardial infarction”, or procedures such as “stent and/or angioplasty of coronary arteries”<br><br>Use of organic nitrates (C01DA) or ranolazine (C01EB18)
Liver disease	Self or proxy-reported diagnosis, including “chronic hepatitis”,” hepatitis B”, “hepatitis C”, “hepatic cirrhosis”
Obesity	Body mass index ≥30 kg/m^2
Osteoarthritis	Self or proxy-reported diagnosis
Osteoporosis	Self or proxy-reported diagnosis<br><br>Use of bisphosphonates (M05BA) or other drugs affecting bone structure and mineralization (M05BX)
Pancreatic disorders	Self or proxy-reported diagnosis<br><br>Use of multienzymes (A09AA02)
Peripheral neuropathy	Self or proxy-reported diagnosis
Prostate disorders	Self or proxy-reported diagnosis, including “benign neoplasm of prostate”, “benign prostatic hyperplasia”, “prostatic adenoma”, or “transurethral prostatectomy” procedure
Sleep disorders	Self or proxy-reported diagnosis
Thyroid disorders	Self or proxy-reported diagnosis, including “hyperthyroidism”, “hypothyroidism”, “thyroid disorder”, or procedures such as “thyroidectomy” or “thyroid operation”<br><br>Use of thyroid therapy (H03) preparations

Upper gastrointestinal disorders	Self or proxy-reported diagnosis, including “chronic gastritis”, “diverticulum oesophageal”, “gastric ulcer”, “gastroesophageal reflux disease”, or procedure such as “gastrectomy” or “gastric polypectomy”
Urinary or bowel incontinence	Self or proxy-reported diagnosis

ATC codes in parentheses

References

1. Calderón-Larrañaga, A. *et al.* Assessing and measuring chronic multimorbidity in the older population: a proposal for its operationalization. *J. Gerontol. A Biol. Sci. Med. Sci.* **72**, 1417–1423 (2017).
2. Schaeffner, E. S. *et al.* Two novel equations to estimate kidney function in persons aged 70 years or older. *Ann. Intern. Med.* **157**, 471–481 (2012).
3. Beridze, G. *et al.* Concordance and discrepancies among 5 creatinine-based equations for assessing estimated glomerular filtration rate in older adults. *JAMA Netw. Open* **6**, e234211 (2023).
4. Folstein, M. F., Folstein, S. E. & McHugh, P. R. “Mini-mental state”. A practical method for grading the cognitive state of patients for the clinician. *J. Psychiatr. Res0* **12**, 189–98 (1975).
5. Radloff, L. S. The CES-D scale: a self-report depression scale for research in the general population. *Appl. Psychol. Meas.* **1**, 385–401 (1977).

Supplementary table 2. Chronic disease prevalence and distribution of the number of chronic diseases in the study population across multimorbidity patterns.

Diseases	**Overall**, N = 1199[1]	**Unspecific**, N = 660[1]	**Respiratory**, N = 88[1]	**Psychiatric**, N = 290[1]	**Cardiometabolic**, N = 161[1]	**p-value**[2]
Dyslipidaemia	1,064 (89%)	587 (89%)	78 (89%)	259 (89%)	140 (87%)	0.9
Hypertension	984 (82%)	542 (82%)	70 (80%)	225 (78%)	147 (91%)	0.003
Osteoarthritis	929 (77%)	498 (75%)	69 (78%)	254 (88%)	108 (67%)	<0.001
CKD	587 (49%)	300 (45%)	37 (42%)	134 (46%)	116 (72%)	<0.001
Obesity	440 (37%)	250 (38%)	38 (43%)	96 (33%)	56 (35%)	0.3
Upper GI disorders	388 (32%)	154 (23%)	41 (47%)	98 (34%)	95 (59%)	<0.001
Incontinence	307 (26%)	140 (21%)	19 (22%)	97 (33%)	51 (32%)	<0.001
Diabetes	270 (23%)	141 (21%)	12 (14%)	32 (11%)	85 (53%)	<0.001
Cognitive impairment	281 (23%)	138 (21%)	29 (33%)	67 (23%)	47 (29%)	0.021
Depression	262 (22%)	0 (0%)	26 (30%)	192 (66%)	44 (27%)	<0.001
Sleep disorders	222 (19%)	0 (0%)	18 (20%)	172 (59%)	32 (20%)	<0.001
Thyroid disease	225 (19%)	117 (18%)	14 (16%)	79 (27%)	15 (9.3%)	<0.001
Anaemia	212 (18%)	85 (13%)	15 (17%)	33 (11%)	79 (49%)	<0.001
COPD	206 (17%)	57 (8.6%)	88 (100%)	28 (9.7%)	33 (20%)	<0.001
Ischemic heart disease	184 (15%)	58 (8.8%)	15 (17%)	26 (9.0%)	85 (53%)	<0.001
Cancer	163 (14%)	84 (13%)	7 (8.0%)	37 (13%)	35 (22%)	0.007
Cerebrovascular disease	164 (14%)	73 (11%)	13 (15%)	33 (11%)	45 (28%)	<0.001
Atrial fibrillation	138 (12%)	49 (7.4%)	10 (11%)	18 (6.2%)	61 (38%)	<0.001
Asthma	103 (8.6%)	7 (1.1%)	88 (100%)	3 (1.0%)	5 (3.1%)	<0.001
Prostate disorders	93 (7.8%)	42 (6.4%)	8 (9.1%)	4 (1.4%)	39 (24%)	<0.001
Osteoporosis	92 (7.7%)	39 (5.9%)	7 (8.0%)	39 (13%)	7 (4.3%)	<0.001
Heart failure	87 (7.3%)	9 (1.4%)	7 (8.0%)	6 (2.1%)	65 (40%)	<0.001
Liver disease	54 (4.5%)	24 (3.6%)	2 (2.3%)	14 (4.8%)	14 (8.7%)	0.048
Cataract	48 (4.0%)	18 (2.7%)	3 (3.4%)	27 (9.3%)	0 (0%)	<0.001
Bradycardias	36 (3.0%)	0 (0%)	2 (2.3%)	3 (1.0%)	31 (19%)	<0.001
Pancreatic disorders	25 (2.1%)	14 (2.1%)	1 (1.1%)	3 (1.0%)	7 (4.3%)	0.14

**Diseases**	**Overall**, N = 1199[1]	**Unspecific**, N = 660[1]	**Respiratory**, N = 88[1]	**Psychiatric**, N = 290[1]	**Cardiometabolic**, N = 161[1]	**p-value**[2]
Arteriosclerosis	20 (1.7%)	8 (1.2%)	5 (5.7%)	2 (0.7%)	5 (3.1%)	0.008
Glaucoma	13 (1.1%)	7 (1.1%)	0 (0%)	2 (0.7%)	4 (2.5%)	0.3
Cardiac valve disease	12 (1.0%)	4 (0.6%)	0 (0%)	3 (1.0%)	5 (3.1%)	0.053
Peripheral neuropathy	7 (0.6%)	6 (0.9%)	0 (0%)	1 (0.3%)	0 (0%)	0.8
Autoimmune diseases	6 (0.5%)	4 (0.6%)	0 (0%)	2 (0.7%)	0 (0%)	>0.9
Allergy	3 (0.3%)	0 (0%)	2 (2.3%)	0 (0%)	1 (0.6%)	0.003
Blindness visual impairment	2 (0.2%)	1 (0.2%)	1 (1.1%)	0 (0%)	0 (0%)	0.3
Gout	2 (0.2%)	1 (0.2%)	0 (0%)	0 (0%)	1 (0.6%)	0.4
Deafness hearing loss	1 (<0.1%)	0 (0%)	0 (0%)	1 (0.3%)	0 (0%)	0.4
Inflammatory bowel disease	1 (<0.1%)	0 (0%)	1 (1.1%)	0 (0%)	0 (0%)	0.073
Epilepsy	0 (0%)	0 (0%)	0 (0%)	0 (0%)	0 (0%)	
Number of chronic conditions	6.00 (5.00, 8.00)	5.00 (4.00, 6.00)	8.00 (7.00, 10.00)	7.00 (5.25, 8.00)	9.00 (7.00, 10.00)	<0.001

[1]n (%); Median (IQR)

[2]Pearson's Chi-squared test; Fisher's exact test; Kruskal–Wallis rank sum test

Supplementary fig. 1. Adjusted Bayesian information criteria (ABIC) across different number of latent classes.

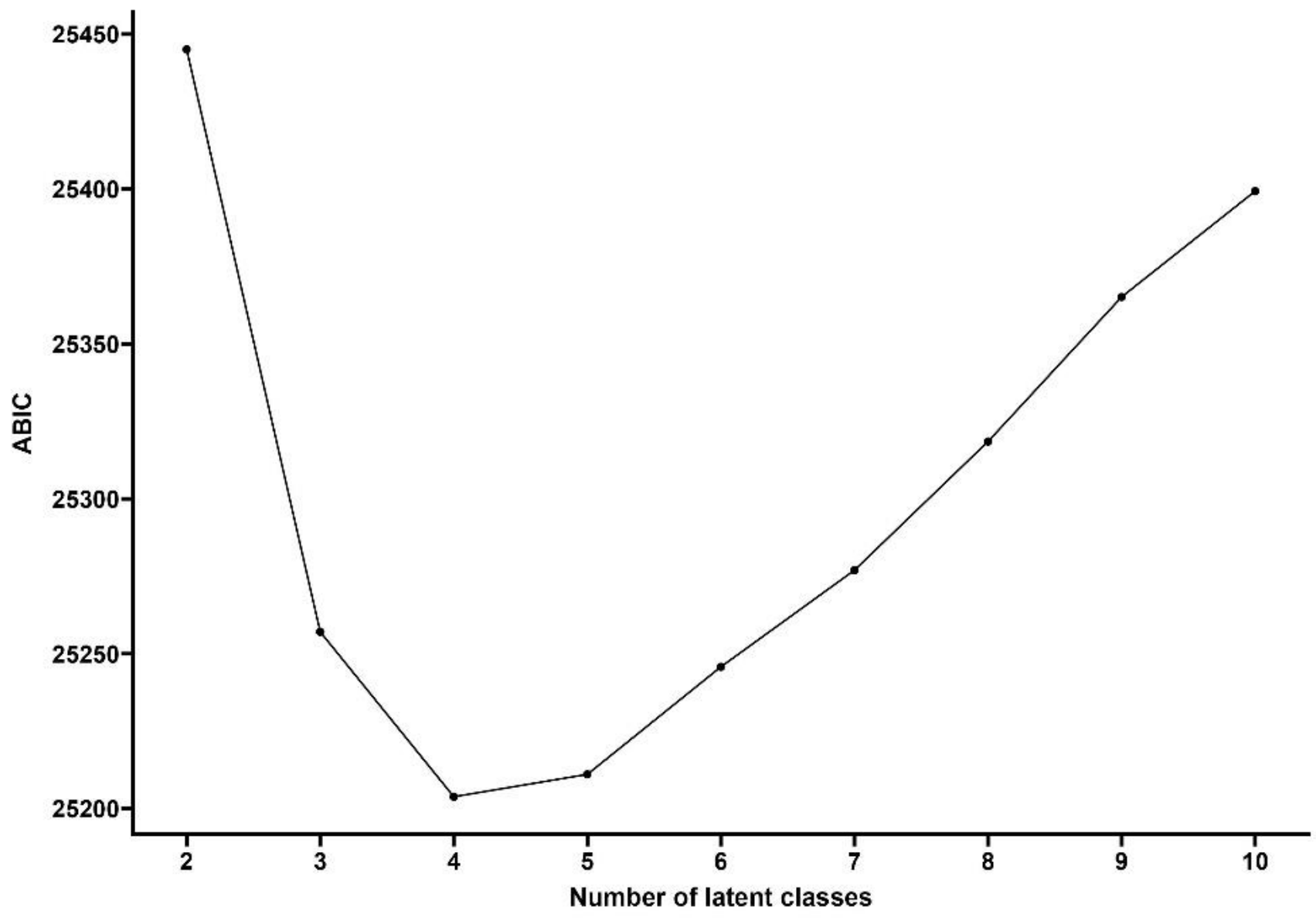

The ABIC curve clearly shows a minimum for the solution with four latent classes corresponding to the four patterns identified as the “Unspecific”, “Respiratory”, “Psychiatric”, and “Cardiometabolic”.

Supplementary fig. 2. Covariate balance before and after inverse probability of treatment weighting (IPTW) across multimorbidity patterns (unspecific, n=660; respiratory, n=88; psychiatric, n=290; cardiometabolic, n=161).

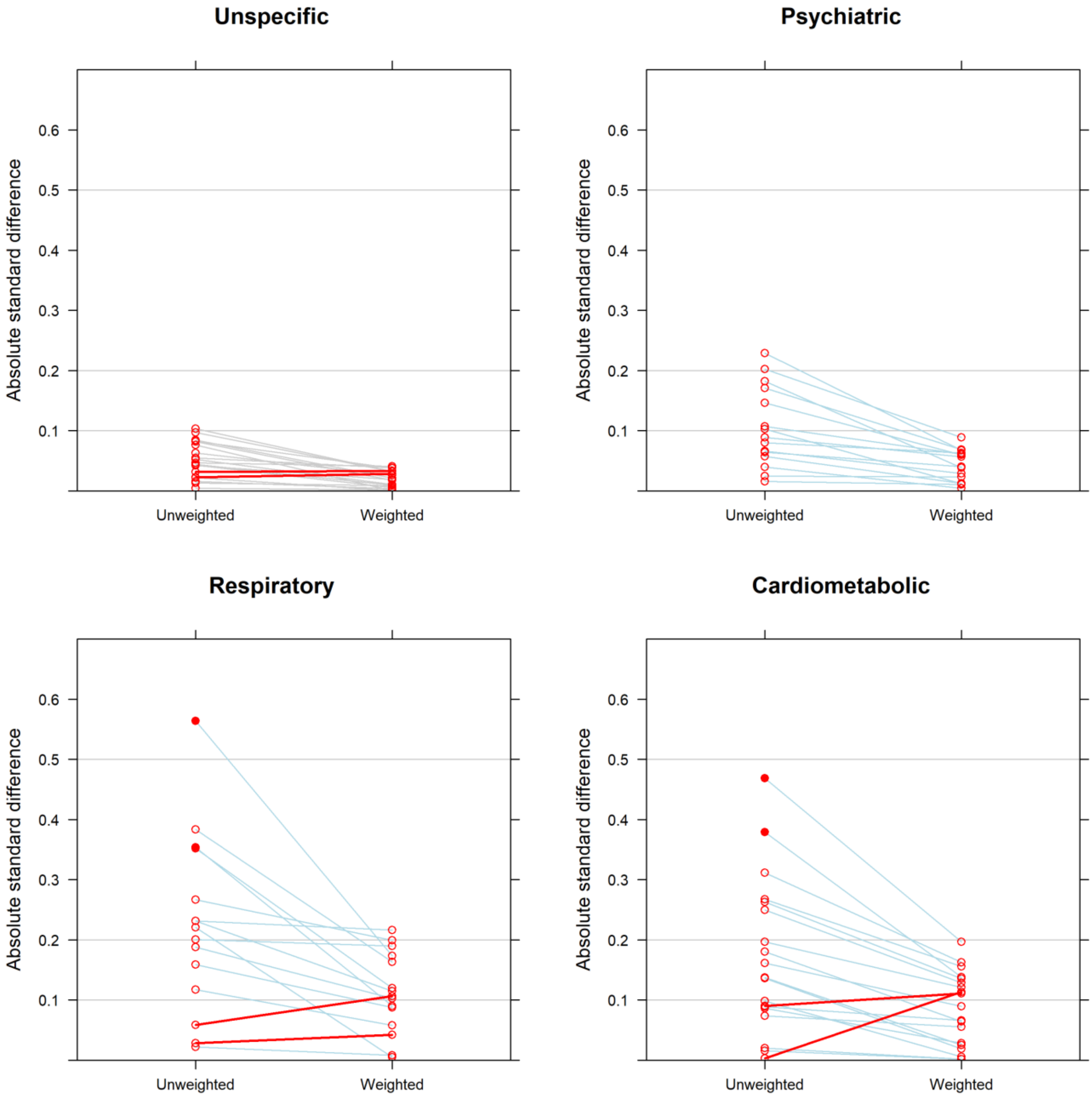

Each panel displays the absolute standardised differences for all baseline covariates before (Unweighted) and after (Weighted) IPTW. Although the multimorbidity patterns show varying degrees of initial imbalance, weighting substantially improves balance across all covariates. After IPTW, the absolute standardised differences are reduced to approximately 0.2 or below for all baseline variables, indicating satisfactory balance and supporting the validity of the weighted subgroup analyses.

Supplementary fig. 3. Inverse probability of treatment weighting (IPTW) Kaplan–Meier curves for incident mobility disability or death within each of the multimorbidity patterns.

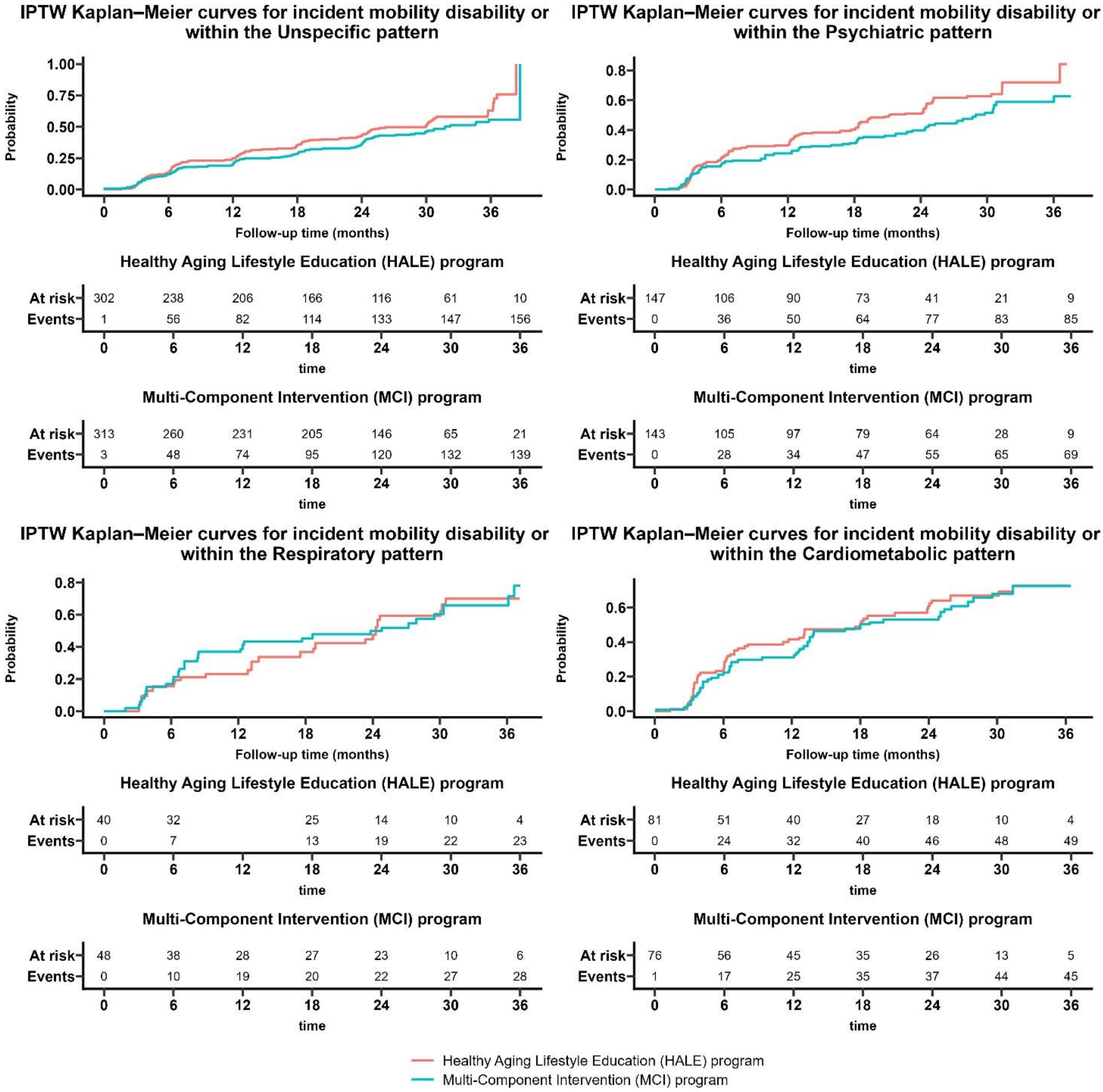

Supplementary fig. 4. Power to detect treatment–pattern interaction across increasing sample sizes in the Monte Carlo simulation calibrated to multimorbidity pattern subgroup analyses in the SPRINTT trial.

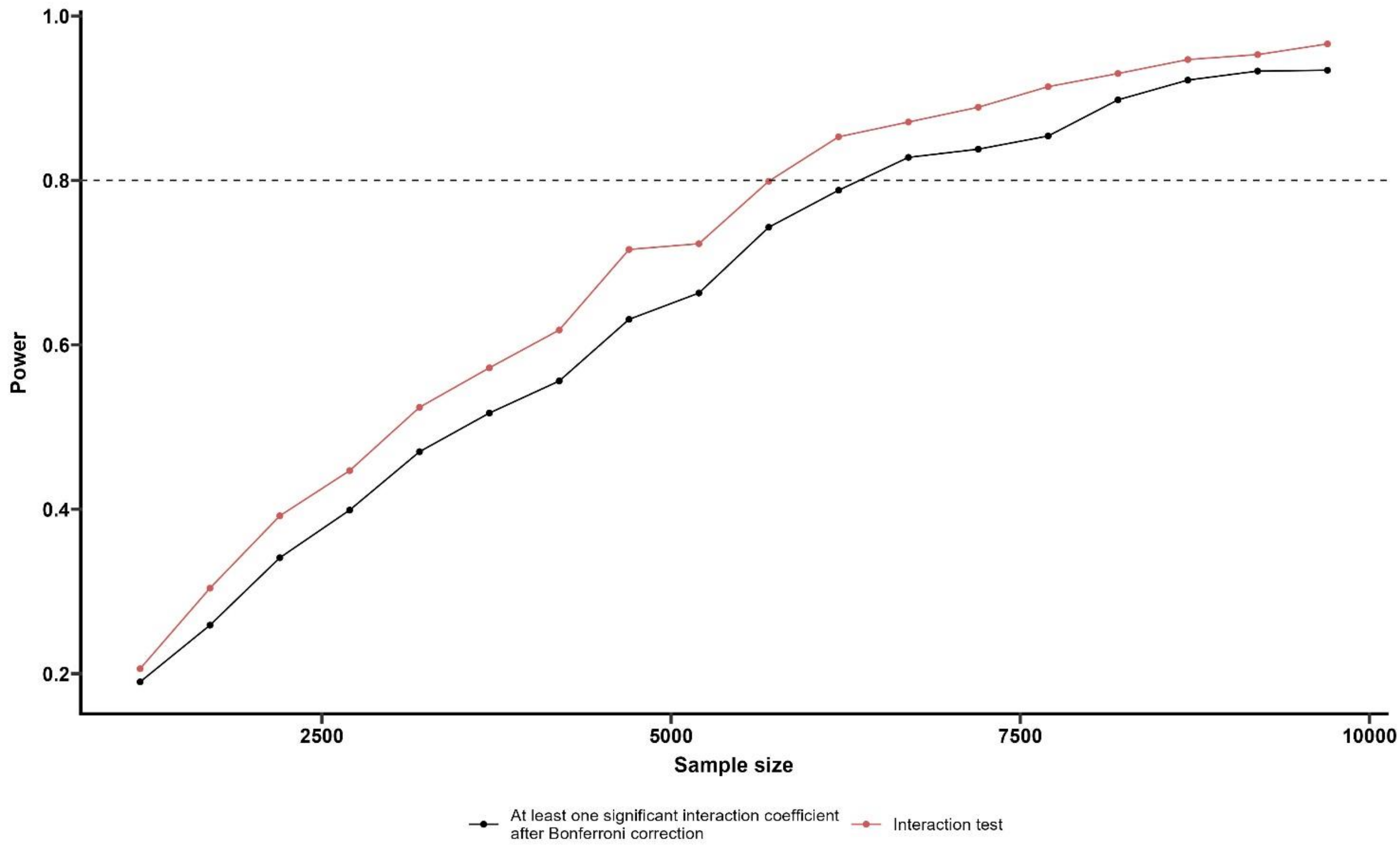

The red curve shows the power of the overall interaction test from a Cox proportional hazards model. The black curve shows the probability of detecting at least one significant subgroup-specific interaction coefficient after Bonferroni correction for multiple comparisons. Both approaches exhibit very low power at sample sizes comparable to the original trial (1199 participants), with the overall interaction test consistently outperforming the Bonferroni-adjusted approach due to its less conservative nature. Power exceeds 80% only at large sample sizes (>6500 participants), illustrating that realistic heterogeneity in treatment effects would be likely difficult to detect at the observed study size.
