# Supplementary material for: Multimorbidity patterns influence mobility disability prevention in frail older adults from the SPRINTT trial
Source: Nat Aging. 2026 Aug 31;6(9):1967–78. doi: 10.1038/s43587-026-01188-x (PMC13577888; doi:10.1038/s43587-026-01188-x)
Supplement: Supplementary file 2 [file 43587_2026_1188_MOESM2_ESM.pdf]

nature portfolio

Corresponding author(s): Emanuele Marzetti

Last updated by author(s): May 7, 2026

Reporting Summary

Nature Portfolio wishes to improve the reproducibility of the work that we publish. This form provides structure for consistency and transparency in reporting. For further information on Nature Portfolio policies, see our Editorial Policies and the Editorial Policy Checklist.

Statistics

For all statistical analyses, confirm that the following items are present in the figure legend, table legend, main text, or Methods section.

n/a	Confirmed	
☐	☒	The exact sample size (n) for each experimental group/condition, given as a discrete number and unit of measurement
☐	☒	A statement on whether measurements were taken from distinct samples or whether the same sample was measured repeatedly
☐	☒	The statistical test(s) used AND whether they are one- or two-sided<br>*Only common tests should be described solely by name; describe more complex techniques in the Methods section.*
☐	☒	A description of all covariates tested
☐	☒	A description of any assumptions or corrections, such as tests of normality and adjustment for multiple comparisons
☐	☒	A full description of the statistical parameters including central tendency (e.g. means) or other basic estimates (e.g. regression coefficient) AND variation (e.g. standard deviation) or associated estimates of uncertainty (e.g. confidence intervals)
☐	☒	For null hypothesis testing, the test statistic (e.g. F, t, r) with confidence intervals, effect sizes, degrees of freedom and P value noted<br>*Give P values as exact values whenever suitable.*
☐	☒	For Bayesian analysis, information on the choice of priors and Markov chain Monte Carlo settings
☒	☐	For hierarchical and complex designs, identification of the appropriate level for tests and full reporting of outcomes
☒	☐	Estimates of effect sizes (e.g. Cohen's d, Pearson's r), indicating how they were calculated

Our web collection on statistics for biologists contains articles on many of the points above.

Software and code

Policy information about availability of computer code

Data collection	All data were collected through a CleanWEB-based software compliant with international GCP and cGMP standards
Data analysis	All analyses were conducted in R (version 4.5.2)

For manuscripts utilizing custom algorithms or software that are central to the research but not yet described in published literature, software must be made available to editors and reviewers. We strongly encourage code deposition in a community repository (e.g. GitHub). See the Nature Portfolio guidelines for submitting code & software for further information.

Data

Policy information about availability of data

All manuscripts must include a data availability statement. This statement should provide the following information, where applicable:

- Accession codes, unique identifiers, or web links for publicly available datasets
- A description of any restrictions on data availability
- For clinical datasets or third party data, please ensure that the statement adheres to our policy

Anonymised raw trial data can be shared on request to Luca Mariotti (luca.mariotti1@unicatt.it). A data access agreement needs to be signed

Research involving human participants, their data, or biological material

Policy information about studies with human participants or human data. See also policy information about sex, gender (identity/presentation), and sexual orientation and race, ethnicity and racism.

Reporting on sex and gender	Information on sex was collected in the SPRINTT dataset. Gender information was not collected. Analyses were stratified by sex with results reported in tables and figures.
Reporting on race, ethnicity, or other socially relevant groupings	Information on race and ethnicity, living arrangement, marital status, income, and education attainment was collected in the SPRINTT dataset but was not used in the present study.
Population characteristics	Demographic variables, including age and sex, were collected during the pre-randomisation screening. Median age was 79.0 yrs (IQR: 75.0-84.0), and 71% were women. The number of chronic conditions for each participant was derived from diagnoses classified using a stardardised assessment integrating multiple data dources (e.g., medical history interviews, structured questionnaires, anthropometric measurements, vital signs, and laboratory analyses). The median number of chronic conditions was 6.0 (IQR: 5.0-8.0). Baseline characteristics were comparable between intervention groups. Diagnoses were coded according to the International Classification of Diseases, Ninth Revision (ICD-9), and aggregated into 60 chronic disease categories using a validated classification system. Medications were collected and reviewed to support diagnostic adjudication.
Recruitment	Each study site developed their own recruitment plan to suit local conditions. Common strategies included media ads, direct mail, community outreach, and engagement with healthcare providers and senior organizations. Promotional materials were widely distributed, and a dedicated website (www.mysprintt.eu) supported awareness of the project among the general public. A centralised monitoring team tracked recruitment outcomes, shared effective strategies, and supported sites with difficulties through teleconferences, visits, and tailored corrective actions.
Ethics oversight	The study protocol was approved by the Ethics Committee of the Università Cattolica del Sacro Cuore, Rome, Italy (approval no. 15611/15), and subsequently ratified by the ethics committees of all participating institutions. Participation was voluntary, and no financial compensation was provided. Written informed consent was obtained from all participants at screening.

Note that full information on the approval of the study protocol must also be provided in the manuscript.

Field-specific reporting

Please select the one below that is the best fit for your research. If you are not sure, read the appropriate sections before making your selection.

☒ Life sciences ☐ Behavioural & social sciences ☐ Ecological, evolutionary & environmental sciences

For a reference copy of the document with all sections, see nature.com/documents/nr-reporting-summary-flat.pdf

Life sciences study design

All studies must disclose on these points even when the disclosure is negative.

Sample size	Calculation of sample size for the SPRINTT trial was based on the LIFE study (NCT01072500), which showed that physical activity reduced mobility disability in participants with SPPB < 8, the trial aimed to enroll 1,200 participants with SPPB 3–7. This provided 85% power to detect a 25% reduction in mobility disability risk over 36 months (assuming 434 events, 25% dropout over two years, and a two-sided $\alpha = 0.05$). An exploratory cohort of 300 participants with SPPB 8–9 was added to broaden the functional spectrum, its size determined by feasibility and resources. For the present analysis, only randomised participants with baseline SPPB 3–7 and multimorbidity (≥ 2 chronic conditions) were included. No formal sample size estimation was performed for this study.
Data exclusions	A total of six participants were excluded from the analysis, as they exhibited fewer than two comorbid conditions, not fulfilling the clinical criterion for multimorbidity adopted in our analyses.
Replication	Study results were not replicated in independent cohorts. Data are available from the corresponding author upon reasonable request for replication by qualified researchers.
Randomization	Participants were randomised in a 1:1 ratio to either the multicomponent intervention or the lifestyle education control group, using a web-based randomisation system implementing a permuted block algorithm. Randomisation was stratified by study site, and site.
Blinding	An evaluator-blinded design was used to preserve trial integrity. Outcome assessors were unaware of group allocation, clinic and laboratory results, and participant adherence to the assigned intervention.

Reporting for specific materials, systems and methods

We require information from authors about some types of materials, experimental systems and methods used in many studies. Here, indicate whether each material, system or method listed is relevant to your study. If you are not sure if a list item applies to your research, read the appropriate section before selecting a response.

Materials & experimental systems

n/a	Involved in the study
☒	☐ Antibodies
☒	☐ Eukaryotic cell lines
☒	☐ Palaeontology and archaeology
☒	☐ Animals and other organisms
☐	☒ Clinical data
☒	☐ Dual use research of concern
☒	☐ Plants

Methods

n/a	Involved in the study
☒	☐ ChIP-seq
☒	☐ Flow cytometry
☒	☐ MRI-based neuroimaging

Clinical data

Policy information about clinical studies

All manuscripts should comply with the ICMJE guidelines for publication of clinical research and a completed CONSORT checklist must be included with all submissions.

Clinical trial registration	ClinicalTrials.gov NCT02582138
Study protocol	The full study protocol is accessible at https://www.bmj.com/content/bmj/suppl/2022/05/11/bmj-2021-068788.DC1/berr068788.ww.pdf
Data collection	Data were collected at study centres. Recruitment took place between January 2016 and November 2017, and the last follow-up visit was conducted on 31 October 2019.
Outcomes	The primary outcome was incident mobility disability, assessed using the 400 m walk test. Participants were instructed to complete ten laps of a 20 m course at their usual pace, avoiding overexertion. The test was administered three months after randomisation and every six months thereafter. When the test could not be performed, a predefined adjudication process was applied. Mobility disability was assigned if gait speed over 4 m was equal or below 0.4 m/s, or >0.4 m/s with the use of a walking aid other than a single cane, or based on medical records and self- or proxy-reported walking disability. Cases not meeting these criteria were reviewed by an independent adjudication committee, which evaluated clinical data, functional assessments, and adverse event reports. Participants were censored at their last succesful test if disability criteria were not met at the end of the trial, at the first visit with a gap >9 months between two succesful tests, or at randomisation if no post-baseline test was available. Mobility disability was defined as occurring on the date when a participant failed the 400 m walk test, was unable or unwilling to attempt it, and adjudicated as disabled, was unable to attempt without adjudication, or died.

Plants

Seed stocks	*Report on the source of all seed stocks or other plant material used. If applicable, state the seed stock centre and catalogue number. If plant specimens were collected from the field, describe the collection location, date and sampling procedures.*
Novel plant genotypes	*Describe the methods by which all novel plant genotypes were produced. This includes those generated by transgenic approaches, gene editing, chemical/radiation-based mutagenesis and hybridization. For transgenic lines, describe the transformation method, the number of independent lines analyzed and the generation upon which experiments were performed. For gene-edited lines, describe the editor used, the endogenous sequence targeted for editing, the targeting guide RNA sequence (if applicable) and how the editor was applied.*
Authentication	*Describe any authentication procedures for each seed stock used or novel genotype generated. Describe any experiments used to assess the effect of a mutation and, where applicable, how potential secondary effects (e.g. second site T-DNA insertions, mosiacism, off-target gene editing) were examined.*
